# Supplementary material for: Seroconversion of a Swine Herd in a Free-Range Rural Multi-Species Farm against HPAI H5N1 2.3.4.4b Clade Virus
Source: Microorganisms. 2023 Apr 28;11(5):1162. doi: 10.3390/microorganisms11051162 (PMC10224318; doi:10.3390/microorganisms11051162)
Supplement: Supplementary file 1 [file microorganisms-11-01162-s001.zip › microorganisms-2340149-supplementary.pdf]

Supplementary table 1 – Serological data testing swine sera by the HI, MN and ELISA assays.

			HI assay					MN assay	
	**Serum ID**	**Anti-NP ELISA (S/N %)** [a]	**H1N1 sw/IT/6831 (avian-like)**	**H1N2 sw/IT/166853 (human-like)**	**H1N1 Italy/2810 (pandemic)**	**H3N2 sw/IT/8088 (human-like)**	**H5N1 ew/IT (clade 2.3.4.4b)**	**H5N1 ew/IT (clade 2.3.4.4b)**	
	9437/1	31,8	NEG[b]	NEG	NEG	NEG	NEG	N.D.[c]	
	9437/2	14,9	NEG	NEG	NEG	NEG	NEG	N.D.	
	9437/3	19,5	NEG	NEG	NEG	NEG	NEG	N.D.	
	9437/5	6,1	NEG	1:80	NEG	NEG	1:320	1:320	
	9437/6	39,2	NEG	NEG	NEG	NEG	NEG	N.D.	
	9437/7	10,1	NEG	1:20	NEG	NEG	1:20	N.D.	
	9503/1	20,1	NEG	1:80	NEG	NEG	1:160	1:160	
	9503/4	11,1	NEG	NEG	NEG	NEG	1:20	N.D.	
	9503/5	9,6	NEG	NEG	NEG	NEG	NEG	N.D.	
	9503/7	5,8	NEG	NEG	NEG	NEG	NEG	N.D.	
	9503/8	20,1	NEG	NEG	NEG	NEG	1:640	1:320	
	9503/9	31,3	NEG	NEG	NEG	NEG	NEG	N.D.	
	9503/11	22,8	NEG	NEG	NEG	NEG	NEG	N.D.	
	9503/12	5,2	NEG	1:80	NEG	NEG	1:40	N.D.	
	9503/13	10,9	NEG	1:20	NEG	NEG	1:80	N.D.	
	9503/15	5	NEG	NEG	NEG	NEG	NEG	N.D.	
	9503/17	23,9	NEG	NEG	NEG	NEG	1:40	N.D.	
	9503/18	21,4	NEG	NEG	NEG	NEG	1:80	N.D.	
	9503/20	7,9	NEG	NEG	NEG	NEG	1:320	1:320	
	9503/21	17,3	NEG	1:320	NEG	NEG	1:320	1:640	
	9503/22	21,5	NEG	NEG	NEG	NEG	1:40	N.D.	
	9503/23	7,8	NEG	NEG	NEG	NEG	1:1280	1:320	
	9503/24	16,9	NEG	NEG	NEG	NEG	1:180	N.D.	
	9503/25	20,8	NEG	NEG	NEG	NEG	1:10	N.D.	
	9503/26	10,3	NEG	NEG	NEG	NEG	1:80	N.D.	
	9503/29	37,1	NEG	NEG	NEG	NEG	NEG	N.D.	
	9503/30	42,5	NEG	NEG	NEG	NEG	1:10	N.D.	
	9503/34	16,2	NEG	NEG	NEG	NEG	NEG	N.D.	
	9503/35	37,1	NEG	NEG	NEG	NEG	NEG	N.D.	
	9503/36	6,5	NEG	NEG	NEG	NEG	1:640	1:320	
	9503/37	5	NEG	NEG	NEG	NEG	1:20	N.D.	
	9503/38	9,7	NEG	NEG	NEG	NEG	1:80	N.D.	
	9503/40	5,4	NEG	NEG	NEG	NEG	1:160	1:160	
	9503/42	14,7	NEG	NEG	NEG	NEG	1:10	N.D.	
	9503/43	15,1	NEG	NEG	NEG	NEG	1:640	1:640	
	9503/44	4,8	NEG	NEG	NEG	NEG	NEG	N.D.	
	9503/45	15,7	NEG	NEG	NEG	NEG	NEG	N.D.	
	9503/46	8	NEG	NEG	NEG	NEG	1:40	N.D.	
	9503/47	20,9	NEG	NEG	NEG	NEG	1:20	N.D.	
	9503/49	8,3	NEG	NEG	NEG	NEG	NEG	N.D.	
	9503/50	11,8	NEG	1:320	NEG	NEG	NEG	N.D.	
	9503/51	5,3	NEG	NEG	NEG	NEG	NEG	N.D.	
	9503/52	10,3	NEG	NEG	NEG	NEG	NEG	N.D.	
	9503/53	7	NEG	NEG	NEG	NEG	NEG	N.D.	
	9503/54	16,1	NEG	NEG	NEG	NEG	1:20	N.D.	
	9503/55	16,8	NEG	NEG	NEG	NEG	1:20	N.D.	
	9503/58	15,7	NEG	1:20	NEG	NEG	1:20	N.D.	
	9503/59	11,4	NEG	1:20	NEG	NEG	1:20	N.D.	
	9503/60	10,4	NEG	1:20	NEG	NEG	1:20	N.D.	

a Positive S/N% values ≤ 45, doubtful values in the range 45-50, negative values >50.

b NEG: titers <1:10

c N.D.: not done
